# Automating Construction of Machine Learning Models With Clinical Big Data: Proposal Rationale and Methods

**DOI:** 10.2196/resprot.7757

**Published:** 2017-08-29

**Authors:** Gang Luo, Bryan L Stone, Michael D Johnson, Peter Tarczy-Hornoch, Adam B Wilcox, Sean D Mooney, Xiaoming Sheng, Peter J Haug, Flory L Nkoy

**Affiliations:** ^1^ Department of Biomedical Informatics and Medical Education University of Washington Seattle, WA United States; ^2^ Department of Pediatrics University of Utah Salt Lake City, UT United States; ^3^ Division of Neonatology Department of Pediatrics University of Washington Seattle, WA United States; ^4^ Department of Computer Science and Engineering University of Washington Seattle, WA United States; ^5^ Homer Warner Research Center Intermountain Healthcare Murray, UT United States; ^6^ Department of Biomedical Informatics University of Utah Salt Lake City, UT United States

**Keywords:** machine learning, automated temporal aggregation, automatic model selection, care management, clinical big data

## Abstract

**Background:**

To improve health outcomes and cut health care costs, we often need to conduct prediction/classification using large clinical datasets (aka, clinical big data), for example, to identify high-risk patients for preventive interventions. Machine learning has been proposed as a key technology for doing this. Machine learning has won most data science competitions and could support many clinical activities, yet only 15% of hospitals use it for even limited purposes. Despite familiarity with data, health care researchers often lack machine learning expertise to directly use clinical big data, creating a hurdle in realizing value from their data. Health care researchers can work with data scientists with deep machine learning knowledge, but it takes time and effort for both parties to communicate effectively. Facing a shortage in the United States of data scientists and hiring competition from companies with deep pockets, health care systems have difficulty recruiting data scientists. Building and generalizing a machine learning model often requires hundreds to thousands of manual iterations by data scientists to select the following: (1) hyper-parameter values and complex algorithms that greatly affect model accuracy and (2) operators and periods for temporally aggregating clinical attributes (eg, whether a patient’s weight kept rising in the past year). This process becomes infeasible with limited budgets.

**Objective:**

This study’s goal is to enable health care researchers to directly use clinical big data, make machine learning feasible with limited budgets and data scientist resources, and realize value from data.

**Methods:**

This study will allow us to achieve the following: (1) finish developing the new software, Automated Machine Learning (Auto-ML), to automate model selection for machine learning with clinical big data and validate Auto-ML on seven benchmark modeling problems of clinical importance; (2) apply Auto-ML and novel methodology to two new modeling problems crucial for care management allocation and pilot one model with care managers; and (3) perform simulations to estimate the impact of adopting Auto-ML on US patient outcomes.

**Results:**

We are currently writing Auto-ML’s design document. We intend to finish our study by around the year 2022.

**Conclusions:**

Auto-ML will generalize to various clinical prediction/classification problems. With minimal help from data scientists, health care researchers can use Auto-ML to quickly build high-quality models. This will boost wider use of machine learning in health care and improve patient outcomes.

## Introduction

### Barriers in Using Machine Learning to Realize Value From Clinical Big Data

#### Overview

To improve health outcomes and trim health care costs, we often need to perform predictions/classifications using large clinical datasets (aka, clinical big data), for example, to identify high-risk patients for preventive interventions. Machine learning has been proposed as a key technology for doing this. Machine learning studies computer algorithms, such as support vector machine, random forest, neural network, and decision tree, that learn from data [1]. Trials showed machine learning was used to help the following: (1) lower 30-day mortality rate (odds ratio [OR]=0.53) in emergency department (ED) patients having community-acquired pneumonia [2]; (2) increase on-target hemoglobin values by 8.5%-17% and reduce cardiovascular events by 15%, hospitalization days by 15%, blood transfusion events by 40%-60%, expensive darbepoetin consumption by 25%, and hemoglobin fluctuation by 13% in end-stage renal disease patients on dialysis [3-6]; (3) reduce ventilator use by 5.2 days and health care costs by US $1500 per patient at a hospital respiratory care center [7]; and (4) lower health care costs in Medicare patients’ last 6 months of life by 4.5% [8].

Machine learning could support many clinical activities, but only 15% of hospitals use it for even limited purposes [9]. Compared to statistical methods like logistic regression, machine learning poses less strict assumptions on distribution of data, can increase prediction/classification accuracy, in certain cases doubling it [10-12], and has won most data science competitions [13]. Historically, machine learning was blamed for being a black box. A recent method can automatically explain any machine learning model’s classification results with no accuracy loss [14,15]. Yet, two hurdles remain in using machine learning in health care. First, despite familiarity with data, health care researchers often lack machine learning expertise to directly use clinical big data. Data scientists take years of training to gain deep machine learning knowledge. Health care researchers can work with data scientists, but it takes time and effort for both parties to communicate effectively. Facing a shortage in the United States of data scientists estimated as high as 140,000+ by 2018 [16] and hiring competition from companies with deep pockets, health care systems have a hard time recruiting data scientists [17,18]. As detailed below, developing a machine learning model often requires data scientists to spend extensive time on model selection, which becomes infeasible with limited budgets. Second, some health care systems such as Kaiser Permanente, Intermountain Healthcare (IH), University of Washington Medicine (UWM), Columbia University Medical Center, Veterans Health Administration, and University of Utah Health have teams devoted to data cleaning. Health care researchers can obtain cleaned data from these systems’ enterprise data warehouses (EDWs). In other health care systems, one needs to laboriously clean data before applying machine learning. This is often done with the help of database programmers and/or master-level statisticians, who can also help with data preprocessing and are easier to find than data scientists with deep machine learning knowledge. This study addresses the first hurdle and focuses on automating machine learning model selection and temporal aggregation, an important type of data preprocessing.

#### Barrier 1: Data Scientists Are Needed for Choosing Hyper-Parameter Values and Algorithms

Each learning algorithm includes two categories of parameters: hyper-parameters that a machine learning tool user manually sets prior to model training, and normal parameters automatically tuned in training the model (see Table 1). Given a modeling problem such as predicting 30-day hospital readmission, an analyst manually constructs a model as follows. First, select an algorithm from many pertinent ones like the approximately 40 algorithms for classification included in Waikato Environment for Knowledge Analysis (Weka) [19]. Second, set the values of the selected algorithm’s hyper-parameters. Third, train the model to tune the normal parameters of the selected algorithm automatically. In case model accuracy is unsatisfactory, substitute the algorithm and/or hyper-parameter values and then retrain the model, while using some technique to avoid overfitting on the validation set [20-24]. This process is done over and over until the analyst runs out of time, has a model with good accuracy, or cannot improve further. If feature selection is considered, in each iteration the user also needs to choose a feature selection technique from many applicable ones and set its hyper-parameter values, making this process even more complex. Many possible combinations of hyper-parameter values and learning algorithms lead to hundreds to thousands of laborious and manual iterations to construct a model. These iterations need machine learning expertise, are typically done by a data scientist, and become a barrier [25].

**Table 1:** Two learning algorithms and their example normal parameters and hyper-parameters.

Learning algorithm	Example hyper-parameters	Example normal parameters
Support vector machine	Regularization constant C, kernel to use, tolerance parameter, ε for round-off error, a polynomial kernel’s degree	Support vectors and their Lagrange multipliers
Random forest	Number of independent variables to examine at each inner node of a classification and regression tree, number of trees	Threshold value and input variable used at each inner node of a tree

Model accuracy is affected by choice of hyper-parameter values and learning algorithm. Thornton et al [25] demonstrated that for the 39 classification algorithms included in Weka, the impact on model accuracy averages 46% and can be up to 94%. Even considering a few popular algorithms like random forest and support vector machine, the impact is still above 20% on two-thirds of 21 benchmark datasets. The good choice changes by the particular modeling problem. Computer science researchers have investigated methods for automatically searching hyper-parameter values and algorithms [26]. Some methods can reach equal or better results compared to data scientists’ manual tuning [27,28]. But in case a large number of algorithms are examined, efforts like Auto-WEKA [25,29-31], hyperopt-sklearn [28], and MLbase [32,33] cannot effectively handle large datasets in reasonable time.

A hurdle to automatic search is the amount of time needed to assess on an entire dataset a combination of hyper-parameter values and a learning algorithm. On a modern computer, it takes 2 days to train the champion ensemble model that won the Practice Fusion Diabetes Classification Challenge [34] one time on 9948 patients with 133 input or independent variables (aka, features). Even when disregarding ensembles of more than five base models, aborting long-running tests, and greatly limiting the hyper-parameter value search space (eg, allowing no more than 256 decision trees in a random forest), all impacting search result quality, more than 30 minutes are needed to test an average combination on 12,000 rows (ie, data instances) with 784 attributes [35]. To ensure search result quality, automation efforts often test more than 1000 combinations on the whole dataset [35], leading to months of search time. On a dataset with several dozen attributes and several thousand rows, a search can still take several days [25]. In reality, search time could be thousands of times longer even with a computer cluster for five reasons:

1. Model building is iterative. When a collection of clinical attributes yields low model accuracy, the analyst can include other attributes to boost accuracy. Every iteration takes a new search for hyper-parameter values and learning algorithms.

2. Frequently, ensembles of a large number of base models reach higher accuracy. The training time of an ensemble model rises proportionally to the number of base models.

3. Hyper-parameter values over a broad range are often used to achieve higher accuracy. The above champion ensemble model [34] uses 12 base models. Each random forest base model uses at least 15,000 decision trees.

4. Numerous rows, often from multiple health care systems, can reside in a dataset.

5. Numerous attributes (eg, derived from genomic or textual data) can exist in a dataset. In a hospital without genomic data, a model for readmission prediction was built using 195,901 patients and 3956 attributes already [36]. An algorithm’s execution time rises proportionally to the number of attributes at a minimum and often superlinearly with the number of rows. Irrespective of whether search is done manually or automatically, a slow speed in search frequently causes a search to be terminated early, producing suboptimal model accuracy [35].

#### Barrier 2: Data Scientists Are Needed for Temporally Aggregating Clinical Attributes

Numerous clinical attributes are documented over time needing aggregation prior to machine learning (eg, weight at each patient visit is combined to check whether a patient’s weight kept rising in the previous year). An aggregation period and operator pair (eg, increasing trend, average, count, and maximum) need to be specified for every attribute separately to compute an aggregate value. Usually, clinicians designate pairs and data scientists perform computation. Numerous pairs could be clinically meaningful. The ones that produce high accuracy change by the particular modeling problem and are usually not known in advance. Granted a modeling problem, the analyst picks one or more pairs for each attribute manually, then constructs a model. In case model accuracy is unsatisfactory, the analyst substitutes pairs for some attributes and reconstructs the model, while using some technique to avoid overfitting on the validation set [20-24]. This process between data scientists and clinicians is frequently repeated many times and becomes a barrier. No comprehensive aggregation operator list exists, demanding care to not omit effective operators.

#### Barrier 3: Data Scientists Are Needed for Generalizing Models

A model that is built and is accurate in a health care system often performs poorly and needs to be rebuilt for another system [37], with differing patients, practice patterns, and collected attributes impacting model selection [38,39]. This needs data scientists and is a barrier, as a system often needs many models for diverse clinical activities.

As often quoted, McKinsey estimates that proper use of clinical big data can bring more than US $300 billion in value to US health care each year [16]. The achievable value is surely less, but still significant. To realize value from data, we need new approaches to enable health care researchers to directly use clinical big data and make machine learning feasible with limited budgets and data scientist resources.

### Our Proposed Software

To fill the gap, we will (1) finish developing the open source software, Automated Machine Learning (Auto-ML), to efficiently automate model selection for machine learning with clinical big data and validate Auto-ML on seven benchmark modeling problems of clinical importance, (2) apply Auto-ML and novel methodology to two new modeling problems crucial for care management allocation and pilot one model with care managers, and (3) perform simulations to estimate the impact of adopting Auto-ML on US patient outcomes. We hypothesize that adopting Auto-ML will improve outcomes. Conceptually, Auto-ML will be an automated version of Weka [19] supporting automated temporal aggregation. With minimal help from data scientists, health care researchers can use Auto-ML to quickly build high-quality models. This expands the human resource pool for clinical machine learning and aligns with the industry trend of citizen data scientists, where an organization arms its talent with tools to do deep analytics [40]. Auto-ML can greatly reduce the time and cost required of scarce data scientists, busy clinicians, and computing resources in developing models; enable fast turnaround; and facilitate green computing. The faster a high-quality model gets built and deployed, the earlier it can bring outcome improvement. Auto-ML is not used to reach the maximum possible model accuracy in theory, which is hard to do in reasonable time. Instead, Auto-ML is used to quickly build high-quality models. If needed, data scientists and health care researchers can manually fine-tune them further.

Auto-ML will efficiently automate a selection of feature selection techniques, hyper-parameter values, learning algorithms, and temporal aggregation operators and periods. Auto-ML will continuously show, as a function of time given for model selection, forecasted model accuracy as well as expected patient outcomes of model use. If trends are not promising, the user can abort, add more clinical attributes, and restart. Auto-ML is able to operate on a cluster of computers for scalable processing.

### Gaps in Patient Identification for Care Management and Our Proposed Solutions

#### Overview

Aim 1 involves finishing development of Auto-ML. To improve patient identification and outcomes for care management, Aim 2 involves applying Auto-ML to two new modeling problems by doing the following: (1) use a health care system’s incomplete medical (ie, clinical and/or administrative) data to find future high-cost, diabetic patients and (2) use vast attributes in modern electronic medical records to find future hospital users in asthmatic patients.

Widely used for chronic diseases like asthma and diabetes, care management applies early interventions to high-risk patients to avoid high costs and health status decline [41-43]. In the United States, 7.1 million children (9.6%) and 18.7 million adults (8.0%) [44] have asthma [45,46]. Every year, asthma causes 1.8 million ED visits, 439,000 hospitalizations, US $56 billion in health care costs [47], and 3630 deaths [44]. Proper use of care management can cut down asthma exacerbations; trim costs by up to 15%; drop ED visits and hospital admissions and readmissions by up to 40%; and enhance quality of life, treatment adherence, and patient satisfaction by 30%-60% [42,48-54]. This impacts 63% of annual total asthma costs from asthma exacerbations [51,55].

For care management to be effective within resource constraints, we should only enroll patients with the worst prognosis or those anticipated to have the highest costs. Predictive modeling is widely used for care management [56] as the best method for finding high-risk patients [57], but current approaches have two gaps, as discussed below.

#### Scope Gap

Often, a health care system has incomplete medical data on many of its patients, as a patient’s complete data may spread across several health care systems [58,59]. Typical models for predicting a patient’s costs assume complete data [60-62]. A system usually does not apply models to patients on whom it possibly has incomplete data. As future high-cost patients are not found, care management is not used on them. This limits care management’s scope of use to improve outcomes. UWM is seeking a way to fill the gap, notably for patients with diabetes. To do this, we will use a constraint to find patients who tend to get most of their care at UWM, use UWM’s incomplete data to build a model, and apply it to them to facilitate care management.

#### Accuracy Gap

Existing models for predicting hospital use (ie, inpatient stay or ED visit) in asthmatic patients have low accuracy [63-68]. A typical model [65] missed 75% of future hospital users. A total of 78% of patients in the high-risk group chosen by the model did not use hospitals in the next year. Two factors degrade accuracy. First, several dozen risk factors for hospital use in asthma are known, including age, gender, race/ethnicity, asthma medication use, prior health care use, comorbidities (eg, ischemic heart disease, rhinitis, sinusitis, reflux, anxiety-depression, diabetes, cataracts, chronic bronchitis, and chronic obstructive pulmonary disease), allergies, lung function, number of asthma medication prescribers as a measure of continuity of care, health insurance type, lab test results (eg, total serum immunoglobulin E level and eosinophil count), body mass index, smoking status, secondhand smoke exposure, the ratio of controller to total asthma medications, frequency of nonasthma visits, number of procedures, number of diagnoses, number of prescription drug claims, and asthma questionnaire results (eg, frequency of asthma symptom occurrence, interference with normal activity, nighttime awakening, reliever use for symptom control, forced expiratory volume in 1 second [FEV1], peak expiratory flow rate, FEV1/forced vital capacity ratio, asthma control test score, number of exacerbations last year, controller use, asthma-related acute care, asthma trigger reduction, and asthma medication) [55,63,65,67-73]. Yet, a typical model uses fewer than 10 of these risk factors [63-67]. Existing models were built using data from either clinical trials or outdated electronic medical records gathering limited attributes [74]. No published model uses all known risk factors in modern electronic medical records gathering vast attributes [74]. Second, as with many diseases, many attributes predictive of hospital use in asthma have not been found yet. If we could enroll 5% more of future hospital users in care management, we could avoid up to 8780 hospitalizations and 36,000 ED visits for asthma each year. IH is seeking a way to fill the gap. To do this, we will use vast attributes in IH electronic medical records to build a model predicting hospital use in asthma. The attributes will cover many known risk factors for hospital use in asthma and will be used to find new predictive factors.

### Innovation

Our study is innovative for multiple reasons:

1. With the new software that will be built as part of our project, for the first time, health care researchers with limited machine learning knowledge will quickly be able to build high-quality machine learning models with minimal help from data scientists. The cost and time required of data scientists and clinicians in doing machine learning will be greatly reduced. Also, it will become possible to widely use machine learning in health care to realize value from clinical big data and improve patient outcomes. No existing software can greatly cut the long time required of data scientists in building and generalizing models.

2. We will direct care management to more patients needing it more precisely than current approaches. For patients on whom it possibly has incomplete medical data, a health care system usually does not apply predictive models to find candidates for care management. Existing models for predicting hospital use in asthmatic patients were built mainly using a small set of patients (eg, <1000) or attributes (eg, <10), creating a hurdle in finding many predictive attributes and their interactions. Many known risk factors’ predictive power for hospital use in asthma is unused. In contrast, we will expand the set of diabetic adults for whom predictive models and care management can be used. We will use many asthmatic children and attributes to build new, accurate models for hospital use. The attributes will cover many known risk factors for hospital use in asthma and will be used to find new predictive factors. Our approaches to using incomplete data and vast attributes are new, with principles generalizable to many clinical applications.

3. Our software will (1) automatically choose hyper-parameter values, feature selection techniques, and algorithms for a particular machine learning problem faster than existing methods; (2) efficiently and automatically choose operators and periods for temporally aggregating clinical attributes—no such method currently exists; longitudinal data analysis [75] models the dependent variable; in contrast, our temporal aggregation can use any function of independent variables; (3) continuously show, as a function of time given for model selection, estimated patient outcomes of model use and forecasted model accuracy—for the first time, one can obtain feedback continuously throughout automatic model selection; and (4) enable fast turnaround. There is no such software at present.

4. We will systematically compile the first list of regularly used operators for temporally aggregating clinical attributes. The list can be reused for future clinical data analysis studies. Using MapReduce [76] for distributed computing, we will provide the first implementation of many aggregation operators not offered by current big data software such as Hadoop [77] and Spark [78].

5. We will estimate the impact of adopting our automated machine learning software on US patient outcomes in two scenarios; no such estimate has ever been made. Our impact estimation method is new and can be applied to other scenarios and similar software.

In summary, this study is significant in that it makes machine learning feasible with limited budgets and data scientist resources to help realize value from clinical big data and improve patient outcomes. The models that will be built for the two new modeling problems will help improve care management outcomes.

## Methods

### Overview

Auto-ML will be built atop current big data software, enabling it to operate on one computer or a cluster. Built atop the Hadoop distributed file system, Spark [78] is a major open source software system supporting MapReduce [76] for distributed computing. Spark has an accompanying machine learning library, MLlib [79]. Spark is able to perform machine learning more than 100 times quicker than Hadoop [80]. Auto-ML will be built using the Spark package as well as novel techniques to address the current software’s limitations.

### Aim 1

#### Overview

Our first aim is to finish developing Auto-ML to automate model selection for machine learning with clinical big data and validate Auto-ML on seven benchmark modeling problems of clinical importance.

**Figure 1:**
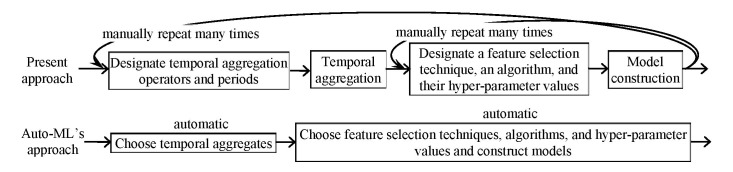
Auto-ML’s approach of constructing machine learning models versus the present one.

Figure 1 compares Auto-ML’s approach of constructing models to the present one. Four steps are carried out sequentially during machine learning: temporally aggregate clinical attributes; choose hyper-parameter values, feature selection techniques, and algorithms; construct models; and assess models. The temporal aggregation step is optional (eg, when no repeatedly recorded attribute exists). Auto-ML will use Spark as the basis for distributed computing. Auto-ML will be coded in Java so it can use the open source software systems Spark and Weka, which all have a Java application programming interface and/or are coded in Java. The user will specify the storage location of the dataset in Auto-ML’s graphical input interface. Auto-ML will then put the dataset into Spark prior to analysis.

**Figure 2:**
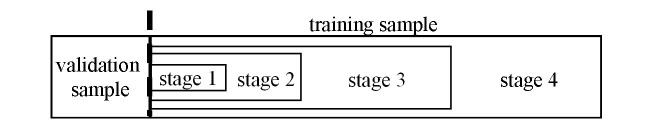
Progressive sampling adopted in our draft automatic model selection method.

#### Auto-ML’s Machine Learning Functions

Auto-ML will integrate MLlib [79] and Weka’s [19] machine learning functions by altering source code and/or invoking the Java application programming interfaces. As a broadly used machine learning tool kit, Weka includes many popular feature selection techniques and learning algorithms. distributedWekaSpark [81] is the distributed computing package of Weka for Spark that is able to operate on a computer cluster. MLlib is a distributed machine learning library in Spark implementing some techniques and algorithms supported by Weka. Auto-ML will support all techniques and algorithms available in Weka. Whenever possible, Auto-ML will use MLlib’s code, which fuses with Spark better than distributedWekaSpark’s code [81].

Weka’s [19] graphical user interface covers feature selection (optional), model construction, and model assessment. In the input interface, the Weka user designates the dependent variable, independent variables, data file, learning algorithm, and the hyper-parameter values of the algorithm. After the user clicks the start button, Weka constructs a model and shows its performance measures. For machine learning, Auto-ML’s graphical user interface will work similarly with two main differences. First, in Weka, the user must specify an algorithm prior to model building. Like Auto-WEKA [25], Auto-ML will use a hyper-parameter to represent the option of feature selection technique and automatically select the hyper-parameter values, technique, and algorithm. The user may override the choice of Auto-ML. Second, to facilitate the user in tracking the automatic selection’s progress, Auto-ML shows a curve presenting the highest accuracy reached over time. The user can terminate the process at any moment and obtain the most accurate model built. In the following sections, we outline the main techniques that we will use to build Auto-ML.

### Aim 1 (a)

#### Overview

For Aim 1 (a), we aim to devise a method to efficiently and automatically choose hyper-parameter values, feature selection techniques, and algorithms. Our review paper [26] showed that few automatic selection methods [25,28-31,82] have been fully implemented and can manage an arbitrary number of combinations of hyper-parameter values and many learning algorithms. All of these methods are similar to or based on the Auto-WEKA automatic selection approach [25], yet none of them can efficiently handle large datasets. To overcome the current methods’ inefficiencies, we drafted a method based on Bayesian optimization for response surface to rapidly identify, for a specific modeling problem, a good combination of hyper-parameter values, a feature selection technique, and a learning algorithm when a large number of algorithms and techniques are examined [35,83]. The method represents the option of technique as a special hyper-parameter; proceeds in stages; and conducts progressive sampling [84], filtering, as well as fine-tuning to rapidly shrink the search space. We conduct fast trials on a small sample taken from the dataset to drop unpromising combinations early, reserving resources to fine-tune promising ones. A combination is promising when a model built using the combination and the sample reaches an error rate below a beginning threshold. Then, we decrease the threshold, enlarge the sample, test and adjust combinations, and cut the search space several times. At the last stage, we find an effective combination using the full dataset.

More specifically, at each stage our method uses a training sample and a validation sample. They have no overlap and contain data instances randomly chosen from the dataset. We keep the validation sample the same and expand the training sample across stages (see Figure 2). At the first stage, we start from a small training sample. For each learning algorithm, we evaluate a fixed number of random hyper-parameter value combinations, if any, as well as its default one. To evaluate a combination, we use the combination, the training sample, and algorithm to construct a model, then use the validation sample to assess the model’s error rate. We identify and remove unpromising algorithms based on the test results. At each subsequent stage that is not the last one, we enlarge the training sample. For each remaining algorithm, we construct a separate regression model, use a Bayesian optimization for response surface approach to choose several new hyper-parameter value combinations, and test these combinations. We identify and remove additional unpromising algorithms based on the test results. At the last stage, we do some final tests on the full dataset to come up with the ultimate search result.

Our draft method needs further optimization for efficiency and effectiveness. To do this, we will expand the draft method to include multiple optimization techniques: the seven outlined in our design paper [24] and the six described in the following sections.

#### Technique 1

In Technique 1, we will use two validation samples to help avoid overfitting. At each stage except for the last one, our draft method [35,83] uses the same validation sample containing a moderate number of data instances to perform many tests. This could lead to overfitting to the validation sample [20-23] that will misguide future search. To help avoid overfitting, we will use two validation samples of equal size with as little overlap as possible, and reduce the frequency of revealing information about the second validation sample [23]. When the dataset has enough data instances, the two validation samples will have no overlap. For a combination of hyper-parameter values and a learning algorithm, we use the combination and the training sample to construct a model and assess the model’s error rate twice, once on either validation sample. Intuitively, the two error rates would be roughly the same in the absence of overfitting. If the error rate on the first validation sample is higher than a specific threshold (eg, in the top 50% of the error rates on the first validation sample of all combinations tested so far at this stage), we use it as the combination’s error rate estimate. Regardless of its exact value, a high error rate estimate will guide future search to avoid the combination’s neighborhood. If the threshold is not exceeded, we compare the error rate on the first validation sample with that on the second. If the former is not lower than the latter by a certain threshold (eg, 5%), we use the former as the combination’s error rate estimate. Otherwise, we use the latter as the combination’s error rate estimate, as overfitting to the first validation sample is likely to have occurred.

The above approach uses the same two validation samples across different stages. Alternatively, if the dataset contains many data instances, we can use a different validation sample at each stage. Each time we arrive at a new stage, we redo sampling to obtain a new validation sample. This also helps avoid overfitting to the same validation sample that is repeatedly used. We will compare the two approaches and choose the one that performs better.

#### Technique 2

In Technique 2, we will use multiple feature selection techniques concurrently to drop unpromising features early. Feature selection and model building time rises proportionally to the number of features at a minimum. Doing a test is slow when many features exist in the dataset. To tackle this issue, we previously proposed that before doing tests, we apply a feature selection technique to the dataset, or a large sample of it, and rapidly drop features not likely to have high predictive power [24]. Yet, like the “no free lunch” theorem [85] shows, no technique can guarantee good performance in all cases. Relying on a single technique can be risky, causing predictive features to be dropped erroneously. To reduce the risk, we will use multiple techniques concurrently. A feature is dropped only if at least a certain number of these techniques all regard it as unpromising.

#### Technique 3

In Technique 3, at the first stage for each learning algorithm, we will ensure a minimum number of tests conducted on every feature evaluator and feature search method. Every feature selection technique adopts a feature evaluator as well as a feature search method [25]. At the first stage for no learning algorithm, our draft method guarantees the number of tests conducted on every feature evaluator or feature search method. Without enough tests, we cannot tell how well a feature evaluator or feature search method works with the algorithm. To tackle this issue, at the first stage for each algorithm, we will check the number of tests conducted on every feature evaluator and feature search method. If the number for a feature evaluator or feature search method is smaller than a specific threshold (eg, 3), we will conduct more tests for the feature evaluator or feature search method to make up the difference. This approach can be adopted for several other components of a data analytic pipeline [86], such as handling imbalanced classes and missing values.

#### Technique 4

In Technique 4, we will share information on the best few results obtained so far among different learning algorithms. Our draft method conducts a separate set of tests for every algorithm. When conducting tests for an algorithm, we may find a combination of a feature selection technique and its hyper-parameter values with superior performance. Yet, the combination may not be tested together with other algorithms, as its information is not shared with them. This can degrade the ultimate search result’s quality. To tackle this issue, we will share information on the best few results obtained so far among different algorithms. At the end of each stage except for the last one, we will identify a prechosen number *n*_1_ (eg, 3) of combinations of algorithms, techniques, and hyper-parameter values that achieve the lowest error rates among all combinations examined so far. Then we will extract the corresponding *n*_2_ combinations of techniques and their hyper-parameter values. Typically, *n*_2_ is equal to *n*_1_. Occasionally, *n*_2_ can be smaller than *n*_1_, as the same combination of a technique and its hyper-parameter values may appear in more than one of the *n*_1_ combinations. At the next stage, for each remaining algorithm, we ensure each of the *n*_2_ combinations of techniques and their hyper-parameter values is tested by adding additional tests, if needed.

#### Technique 5

In Technique 5, for a dataset with relatively few data instances, we will dynamically allocate its data instances between the training and validation samples across stages. A dataset with relatively few data instances can still be large if it contains many features. In this case, our draft method uses a fixed portion of the dataset as the validation sample, which includes a small number of data instances. Because of insufficient testing, the error rate estimates obtained on the trained models can be nonrobust, degrading the ultimate search result’s quality. To tackle this issue, we will dynamically allocate the data instances in the dataset between the training and validation samples across stages. At each stage except for the last one, we give all data instances that are in the dataset, but not in the training sample, to the validation sample. With more data instances in the validation sample, the error rate estimates obtained on the trained models can be more robust. Krueger et al [87] used a similar approach to perform fast cross-validation to select a good hyper-parameter value combination for a given learning algorithm and modeling problem.

#### Technique 6

In Technique 6, we will consider distances between hyper-parameter value combinations when choosing randomly sampled combinations for testing. At each stage that is neither the first nor the final one, for each remaining learning algorithm, our draft method performs one or more rounds of Bayesian optimization. In each round, several new and randomly sampled combinations are chosen out of many for testing and used to adjust the regression model. For the regression model to guide search well, the combinations chosen for testing need to have a reasonable coverage of the hyper-parameter space rather than all reside in a small region. To achieve this, we will attempt to ensure that each randomly sampled combination chosen for testing is separated from each other combination chosen for testing by at least a specific distance. The distance threshold may decrease over stages.

### Aim 1 (b)

For Aim 1 (b), we aim to devise a method to efficiently and automatically choose operators and periods for temporally aggregating clinical attributes. Our design paper [24] outlines our method for automating the process of temporally aggregating clinical attributes. We will flesh out our method’s technical details. Our automation method needs disease-specific knowledge on aggregation operators and periods compiled by clinicians and stored in Auto-ML. Various medical datasets use differing schemas, medical coding systems, and medical terminologies, forming a hurdle in applying precompiled knowledge. To tackle this, the automated temporal aggregation function of Auto-ML demands that the dataset, except for the dependent variable, complies with the Observational Medical Outcomes Partnership (OMOP) common data model [88] and its linked standardized terminologies [89]. Since OMOP standardizes administrative and clinical attributes from 10 or more large US health care systems [90,91], Auto-ML can be adopted for datasets from those systems. We intend to include support for the National Patient-Centered Clinical Research Network (PCORnet) [92] and Informatics for Integrating Biology and the Bedside (i2b2) common data models [93] in the future.

### Aim 1 (c)

**Figure 3:**
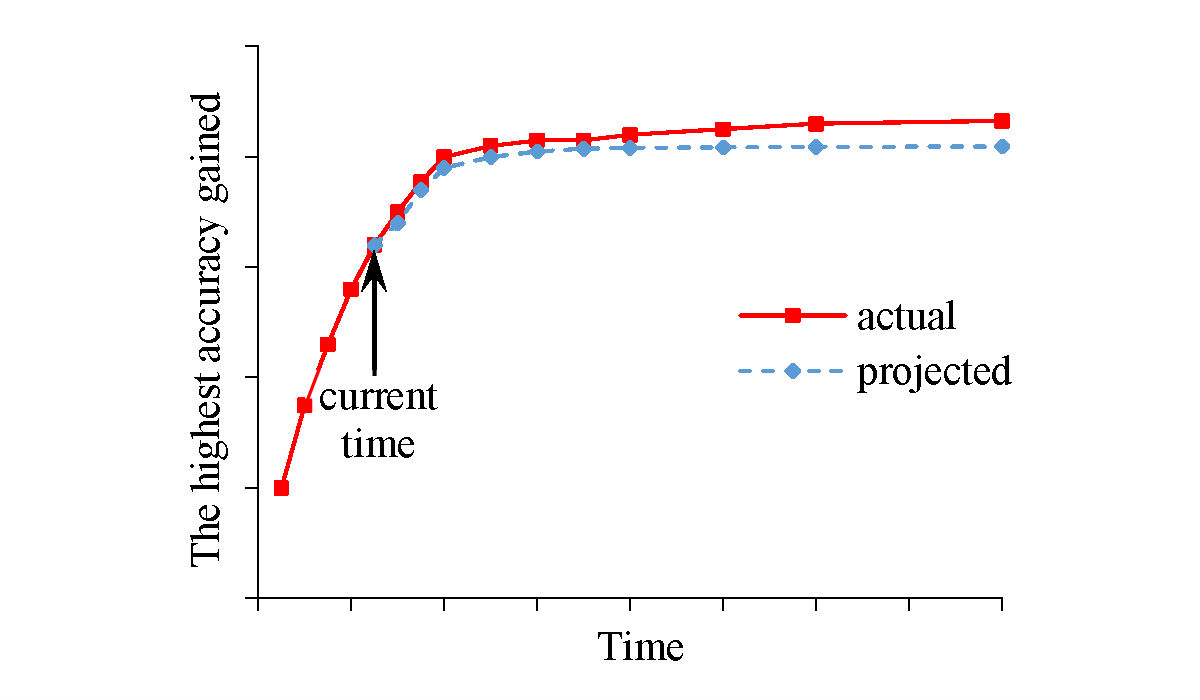
The highest model accuracy gained by Auto-ML over time.

For Aim 1 (c), we aim to continuously show, as a function of time given for model selection, forecasted model accuracy and projected patient outcomes of model use. During automatic selection, to be more useful and user friendly, Auto-ML will show projected patient outcomes of model use and forecasted model accuracy as a function of time given for model selection (see Figure 3). Our design paper [24] outlines our method for doing this. We will flesh out our method’s technical details and write a user manual for Auto-ML.

### Aim 1 (d)

#### Overview

For Aim 1 (d), we aim to validate Auto-ML on seven benchmark modeling problems. We will perform tests with health care researchers using seven modeling problems and datasets that we worked on before. Each problem uses a different dataset from a distinct health care system. We chose these problems because they are related to common diseases, are clinically important, and have readily accessible datasets. Auto-ML can be used for other clinical activities.

#### Subject Recruitment

Via announcements in our institution’s email lists and personal contact, we will recruit 25 health care researchers from UWM, which houses approximately 2500 faculty members, most doing health care research. These health care researchers would regard their familiarity with medical data at the MD level, but would regard their machine learning knowledge as below the level taught in a typical machine learning course for computer science undergraduates. We will conduct purposeful sampling to ensure enough variability [94]. All test participants will have fulfilled UWM’s required training for information security and privacy policy. Participants will receive pseudonyms linking their responses to questions for privacy protection. After giving consent, each will get a copy of the task description, Auto-ML’s user manual, and the metadata document detailing the attributes in the dataset. Upon task completion, each will receive US $200 as compensation for participation.

#### Computing Environment

We will perform all of our experiments on a Health Insurance Portability and Accountability Act (HIPAA)-compliant computer cluster at the University of Washington. After obtaining proper authorization, all test participants and research team members at the University of Washington will be able to access the cluster using their university computers.

#### Modeling Problem 1

Modeling Problem 1 will consist of predicting the diagnosis of type 2 diabetes in adult patients in the next year.

##### Dataset and Patient Population

The clinical and administrative dataset is deidentified and publicly available from the Practice Fusion Diabetes Classification Challenge [15,34], containing 3-year (2009-2012) records as well as the labels of 9948 adult patients from all US states in the following year. A total of 1904 of these patients had a diagnosis of type 2 diabetes in the following year. The dataset comes from an electronic medical record vendor’s EDW; includes repeatedly recorded attributes; and covers patient demographics, allergies, diagnoses, immunizations, medications, smoking status, lab results, and vital signs. We will put this dataset in the OMOP common data model form with its linked standardized terminologies.

##### Model Information

The dependent variable is whether a patient had a diagnosis of type 2 diabetes in the following year. Two-thirds of patients will be randomly selected and put into the training set to construct models. The remaining one-third of patients will form the test set for assessing model performance. We will use the area under the receiver operating characteristic curve (AUC) [19] performance metric.

#### Modeling Problems 2-7

Each of the six problems from Modeling Problems 2-7 uses a distinct, deidentified, and publicly available dataset from the University of California, Irvine machine learning repository [95] to perform a task: (1) Arcene: classify mass spectrometric data into cancer versus normal patterns; (2) Arrhythmia: classify 12-lead electrocardiogram recordings into one of 16 groups about cardiac arrhythmia; (3) Cardiotocography: classify fetal cardiotocograms into one of three fetal states; (4) Diabetic Retinopathy Debrecen: use features obtained from the Messidor image set to detect whether an image includes signs of diabetic retinopathy; (5) Mammographic Mass: use Breast Imaging Reporting and Data System attributes and patient age to separate benign from malignant mammographic masses; (6) Parkinson Speech: use sound recordings to identify Parkinson’s disease patients.

No dataset has repeatedly recorded attributes needing temporal aggregation. The repository [95] includes a detailed description of the problems and datasets. For each dataset, two-thirds of it will be randomly selected and put into the training set to construct models. The remaining one-third will form the test set for assessing model performance. We will use the accuracy metric suitable for multi-class classification.

#### Build Models

We are familiar with the literature on the seven modeling problems. For each problem, our data scientist Dr Luo (GL) will work with the clinicians in our team and manually build a machine learning model with as high accuracy as possible. This accuracy will serve as the gold standard reflecting current best practice of model building. Each of the 25 recruited health care researchers will be randomly given a problem and use Auto-ML to build models for it.

#### Performance Evaluation and Sample Size Justification

We will test the hypothesis that at least 60% of health care researchers can use Auto-ML to achieve model accuracy of at least 95% of the gold standard. When 60% of health care researchers can actually achieve model accuracy of at least 95% of the gold standard, a sample size of 25 health care researchers produces a one-sided 95% lower confidence limit of 42%.

#### User Feedback

When model construction is finished, we will use both open-ended and semistructured questions to survey the 25 health care researchers. As detailed in our design paper [83], we will obtain quantitative outcome measures covering model accuracy, time on task, self-efficacy for constructing machine learning models with clinical big data, satisfaction, trustworthiness, adequacy, and quality of documentation. The questionnaire will contain a text field for gathering comments on Auto-ML. We will refine and finalize Auto-ML by considering suggestions from those comments. We will perform a user satisfaction survey using the System Usability Scale (SUS), a widely used industry standard [96,97] on overall satisfaction ratings for products.

#### Analysis

We will use the accepted inductive approach endorsed by Patton et al [94,98] to do qualitative analysis. We will put the 25 health care researchers’ textual comments into ATLAS.ti Version 8 (ATLAS.ti Scientific Software Development GmbH), a qualitative analysis software tool [99]. The research team will independently highlight quotations related to the issue of using Auto-ML. We will examine quotations, categorize them into precodes, and merge them into categories in multiple iterations. We will synthesize categories to find general themes. Quantitative analyses will include adding the scores in the SUS and presenting every quantitative outcome measure’s descriptive statistics.

### Aim 2

**Overview** Aim 2 involves applying Auto-ML and novel methodology to two new modeling problems crucial for care management allocation, to which our institutions are seeking solutions, and pilot one model with care managers. Both modeling problems use datasets that have repeatedly recorded attributes. We will put the datasets in the OMOP common data model form with its linked standardized terminologies. We will use the same computing environment and recruiting method mentioned in Aim 1 (d). We will recruit two health care researchers not engaged in Aim 1 (d). Each will be randomly given a problem and use Auto-ML to build models for it. Upon task completion, each will receive US $200 as compensation for participation.

#### Modeling Problem 8

Modeling Problem 8 involves the use of vast attributes in modern IH electronic medical records to predict hospital use in asthmatic children in the next year.

##### Patient Population

The patient population consists of IH pediatric patients (0-17 years of age) with asthma in 2005-2016, identified by Schatz et al’s method [63,100,101] as having the following: (1) at least one diagnosis code of asthma according to the International Classification of Diseases, Ninth Revision (ICD-9) (ie, 493.xx), or the International Classification of Diseases, Tenth Revision (ICD-10) (ie, J45/J46.*); or (2) two or more “asthma-related medication dispensings (excluding oral steroids) in a one-year period, including β-agonists (excluding oral terbutaline), inhaled steroids, other inhaled anti-inflammatory drugs, and oral leukotriene modifiers.”

##### Dataset

By running Oracle database Structured Query Language (SQL) queries, our contracted IH data analyst will extract from the IH EDW a deidentified, clinical and administrative dataset, encrypt it, and securely transfer it to a HIPAA-compliant computer cluster for secondary analysis. For each of the last 5 years, the data cover approximately 27,000 asthmatic children. The dataset is the electronic documentation of approximately 95% of pediatric care in Utah [102,103] and includes around 400 attributes partially listed in our paper [14]. These attributes cover many known risk factors for hospital use in asthma and can be used to find new predictive factors.

##### Model Information

The dependent variable is whether an asthmatic patient incurred hospital use—inpatient stay or ED visit—with a primary diagnosis of asthma (ie, ICD-9 493.xx or ICD-10 J45/J46.*) in the following year [14,63,64]. As outcomes need to be computed for the following year, we effectively have 11 years of IH data. We will construct models using the data in the first 10 years and acquire a model’s accuracy estimate via testing on the data in the 11th year. This mirrors future use of the model in practice. We will use the AUC [19] performance metric.

#### Modeling Problem 9

Modeling Problem 9 involves using UWM’s incomplete data to predict individual diabetic adults’ costs in the next year.

##### Patient Population

The patient population includes UWM adult patients (18 years of age or older) with diabetes in 2012-2016, identified by the method in Neuvirth et al [104] as having one or more hemoglobin A1c test results of 6.5% or higher.

##### Dataset

A UWM data analyst will run SQL Server database SQL queries to extract from the UWM EDW a deidentified, clinical and administrative dataset, encrypt it, and securely transfer it to a HIPAA-compliant computer cluster for secondary analysis. The data cover approximately 28,000 diabetic adults per year. Other details of the dataset are similar to those in Modeling Problem 8.

##### Model Information

The dependent variable is a diabetic patient’s total allowed cost to UWM in the following year [60,61]. Allowed costs are less inflated than billed costs and less subject to variation due to member cost sharing than net incurred claims [60]. We will adopt the medical consumer price index [105] to convert all costs to 2016 US dollars to handle inflation. As outcomes need to be computed for the following year, we effectively have 4 years of UWM data. We will construct models using the data in the first 3 years, and acquire a model’s accuracy estimate via testing on the data in the 4th year. This mirrors future use of the model in practice. We will use the *R*^2^ performance metric [61].

To fill the scope gap mentioned in the introduction, we will use a constraint to find patients who tend to get most of their care at UWM. Intuitively, it is easier to identify future high-cost patients among them than among others. We will use UWM’s incomplete data to build a cost prediction model and apply it to them. Regardless of his/her total future cost at non-UWM facilities, a patient who will incur high cost at UWM can be a candidate for care management. By care managing future high-cost patients identified by the model, we will expand the scope of using care management to improve outcomes. The principle of our approach to using incomplete data generalizes to many other clinical applications.

Several candidate constraints exist: (1) the patient had two or more visits to UWM in the past year, (2) the patient has a UWM primary care physician and lives within 5 miles of a UWM hospital, and (3) the patient saw a primary care physician or endocrinologist at UWM in the past year and lives within 60 miles (ie, around 1 hour of driving distance) of a UWM hospital. UWM primary care physicians tend to make referrals within UWM. Endocrinologists often serve some of the same roles as primary care physicians. Usually, a patient incurs high cost because of hospital use. As patients living far away from UWM hospitals are less likely to use them, UWM tends to have less of these patients’ medical data. We will refine the three candidate constraints and investigate others. To select the constraint to be used, we will use PreManage data that UWM has on all of its patients. PreManage is Collective Medical Technologies Inc’s commercial product providing encounter and diagnosis data on inpatient stays and ED visits at many US hospitals [106]. PreManage data cover 105 (approximately 94%) hospitals in Washington, including the four hospitals of UWM. Using UWM data and grouper models like the Clinical Classifications Software system to group diagnosis codes and reduce features [60], we will build two models: one for estimating an inpatient stay’s allowed cost and another for estimating an ED visit’s allowed cost based on patient demographics and diagnosis data. We will use UWM patient demographics data, PreManage diagnosis data, and the two models to estimate the allowed cost of each of a UWM patient’s non-UWM inpatient stays and ED visits reflected by PreManage encounter data. By aggregating the estimated costs of individual non-UWM inpatient stays and ED visits, we will assess each UWM patient’s portion of cost spent at non-UWM hospitals and use the portions to evaluate every candidate constraint. If a health care system does not have enough data to make the two models reasonably accurate, it can use the average costs of an inpatient stay and ED visit to assess each patient’s portion of cost spent at external hospitals. If a system has an insurance plan’s complete claim data on some of its patients, it can use the data similarly.

#### Performance Evaluation and Sample Size Justification

For each of the two new modeling problems, we will test the hypothesis that health care researchers are able to use Auto-ML to achieve higher model accuracy than existing approaches. We will regard Aim 2 as partly successful if we accept the hypothesis in only one problem, and completely successful if we accept the hypothesis in both problems.

For Modeling Problem 8, we will compare the accuracies reached by the model built by the health care researcher and the model in Schatz et al [65]. The first model is built using Auto-ML and vast attributes in modern IH electronic medical records. The second model depicting the existing approach was built using a few known risk factors for hospital use in asthma. Using vast attributes can increase prediction accuracy [107]. We will accept the hypothesis when the first model reaches a higher AUC than the second one by at least .05. Existing predictive models for hospital use in asthma usually achieve an AUC far below .8 [63-68]. Assuming these two models’ prediction results have a correlation coefficient of .6 for both classes and performing a two-sided *Z* test at a significance level of .05, a sample size of 561 data instances per class provides 90% power to find a discrepancy of .05 between the two models’ AUCs. The IH data in the 11th year include about 27,000 asthmatic children, offering enough power to test our hypothesis. Using many patients is essential for improving prediction accuracy, although only a small sample size is needed to show statistical significance.

For Modeling Problem 9, we will compare the accuracies gained by two models. The patient cohort includes those satisfying the chosen constraint. The first model is built by the health care researcher using Auto-ML and clinical and administrative data. The second model depicting the existing approach is a commercial claims-based one available at UWM achieving an *R*^2^ less than 20%. Although the second model was not designed for such use, we will apply it to the patient cohort on whom UWM possibly has incomplete data, which is better than the normal practice of making no predictions. Adding clinical data can increase prediction accuracy [108]. We will accept the hypothesis when the first model reaches a higher *R*^2^ than the second one by at least 5%. Using an *F* test at a significance level of .05 and under the assumption of the existence of 20 features from clinical data in addition to 300 or fewer features used in the second model, a sample size of 443 patients provides 90% power to identify an increase of 5% in *R*^2^ from 20%. Using the second candidate constraint, we estimate that the patient cohort will cover approximately 22% of diabetic adult patients at UWM. The 4th year’s UWM data include approximately 28,000 diabetic adults, offering enough power to test our hypothesis.

#### Pilot With Care Managers

We will pilot the model the health care researcher will build for Modeling Problem 9 with UWM care managers. As a UWM operational project, we are working on this modeling problem and have access to around 25 UWM care managers. Via announcing in their email lists and personal contact, we will recruit five care managers. We will conduct purposeful sampling to ensure enough variability [94]. All test participants will give consent and have fulfilled UWM’s required training for information security and privacy policy. Participants will receive pseudonyms linking their responses to questions for privacy protection. Upon task completion, each will receive US $200 as compensation for participation.

We will use our previously developed method [15] to automatically explain the model’s prediction results. For each care manager, we will randomly select 20 UWM diabetic adult patients, half of whom the model predicts will incur a cost of more than US $30,000. The care manager is unaware of any of these patients’ outcomes in the next year. For each patient, we will first show the care manager the historical, deidentified patient attributes, then show the prediction result and automatically generated explanations, and finally survey him/her using both open-ended and semistructured questions. The questions will cover whether the prediction result and explanations will change his/her enrollment decision on the patient, their usefulness, and their trustworthiness as shown in Table 2. The questionnaire will contain a text field for gathering comments. We will analyze collected information in a similar way to Aim 1 (d).

**Table 2:** The dependent variable list.

Variable	Description
Impact on enrollment decision	Response to the following question: Will the prediction result and automatically generated explanations change your enrollment decision on the patient?
Usefulness of the prediction result	Response to the following question: How useful is the prediction result? Rating is on a 7-point Likert scale, ranging from “not at all” (1) to “very useful” (7).
Usefulness of the automatically generated explanations	Response to the following question: How useful are the automatically generated explanations? Rating is on a 7-point Likert scale, ranging from “not at all” (1) to “very useful” (7).
Trustworthiness of the prediction result	Response to the following question: In your opinion, how much clinical sense does the prediction result make? Rating is on a 7-point Likert scale, ranging from “not at all” (1) to “completely” (7).
Trustworthiness of the automatically generated explanations	Response to the following question: In your opinion, how much clinical sense do the automatically generated explanations make? Rating is on a 7-point Likert scale, ranging from “not at all” (1) to “completely” (7).

For Modeling Problem 8, medication order and refill information is needed for identifying asthma. The IH dataset contains this because IH has its own health insurance plan. If too much refill information is missed at IH, data from the all-payer claims database [109] will be used. For Modeling Problem 9, in our ongoing UWM operational project, we have used around 30 attributes and approximately 6000 patients to build a basic cost prediction model, which achieved an *R*^2^ close to that of the commercial claims-based model. Since the health care researcher will use many more attributes and patients that should increase model accuracy, we expect the cost prediction model built by him/her to achieve a higher *R*^2^ than the claims-based model.

Although using a constraint to fill the scope gap partially addresses UWM data’s incompleteness, UWM still has incomplete medical data on some of its patients satisfying the constraint. For each such diabetic patient, the dependent variable of the patient’s total allowed cost to UWM is only part of the patient’s total allowed cost to all systems. The patient’s features are computed from incomplete data. Both factors may create difficulty for significantly improving *R*^2^. If this occurs, we will revise the dependent variable to a diabetic patient’s total allowed cost to UWM or reflected by PreManage data. On average, the revised dependent variable is closer to the patient’s total allowed cost to all systems than the original one. Recall that based on UWM patient demographics and PreManage diagnosis data, we will use two models to estimate the allowed cost of each of the patient’s non-UWM inpatient stays and ED visits reflected by PreManage encounter data. We will supplement UWM data with PreManage data to make patient data more complete for computing patient features. This approach of using PreManage data and revising the dependent variable can be adopted to improve the accuracy of predicting future hospital use.

For either new modeling problem, if one health care researcher fails to build a reasonably accurate model, we will recruit another health care researcher.

### Aim 3

#### Overview

Aim 3 involves performing simulations to estimate the impact of adopting Auto-ML on US patient outcomes. To determine Auto-ML’s value for future clinical deployment, we will estimate the impact of adopting Auto-ML on US patient outcomes. Trials showed that machine learning helped drop the 30-day mortality rate in ED patients with community-acquired pneumonia (risk ratio≈OR=0.53, as the mortality rate is much less than 1) [2] and cut hospitalization days by 15% in end-stage renal disease patients on dialysis [3]. We will use these two scenarios to demonstrate our simulation method. Our method generalizes to other scenarios and similar software. We will use the same computing environment mentioned in Aim 1 (d). We first discuss the scenario of ED patients with community-acquired pneumonia.

#### Estimate Outcomes

The outcome is 30-day mortality. We will use the latest, deidentified, and publicly available Nationwide Emergency Department Sample (NEDS) database [110], including visit information from approximately 20% of US EDs. Consider the case with Auto-ML. The likelihood, *L*, that an ED can successfully use machine learning for this scenario is equal to *p*_1_× *p*_2_. *p*_1_ is the probability that a health care researcher in the ED can build a high-quality machine learning model for this scenario using Auto-ML. *p*_2_ is the probability that the ED can successfully deploy the model if it can be built. Using Aim 1(d)’s test results on whether health care researchers can use Auto-ML to achieve model accuracy of at least 95% of the gold standard, we will conservatively estimate *p*_1_’s minimum and maximum values (eg, by fitting a normal distribution and using its 2.5 and 97.5 percentile points). Based on his extensive experience with deploying models [2], Dr Haug (PJH) will conservatively estimate *p*_2_’s minimum and maximum values. For each of *p*_1_ and *p*_2_, we will adopt five levels going from the minimum to the maximum value for sensitivity analysis. The middle level is the default one and is used for hypothesis testing.

For each ED in the NEDS database, we will retrieve the annual number of patients with community-acquired pneumonia. We will simulate whether or not the ED can successfully use machine learning for this scenario based on the likelihood, *L*. If success/not success, for each ED patient with community-acquired pneumonia, we will simulate whether the patient will die or not based on the 30-day mortality rate reported in the paper [2] when using/not using machine learning. The overall outcome estimate combines the expected outcomes for all patients and EDs. The patients’ discharge weights in the NEDS database will be used to obtain national estimates from sample data in the database. We will handle the case without Auto-ML similarly by simulating not using machine learning.

#### Outcome Evaluation and Sample Size Justification

Outcomes achieved with and without Auto-ML will be compared. We will test the primary hypothesis that using Auto-ML will be linked to reduced mortality. In the most conservative case assuming a proportion of discordant pairs of 10%, a sample size of 1152 patients provides 90% power to notice an OR of 0.53 [2] using a two-sided McNemar test at a significance level of .05. Each year, community-acquired pneumonia incurs 1.5 million ED patient visits [111], giving adequate power to test the hypothesis. To acquire the whole range of possible outcomes, we will do sensitivity analysis by changing the levels of the probabilities *p*_1_ and *p*_2_, 30-day mortality rate, and rate reduction gained by machine learning.

The scenario of end-stage renal disease patients on dialysis will be handled similarly, with the following main differences. The outcome is number of hospitalization days. The health care unit is dialysis facility. For each US dialysis facility, we will obtain its latest annual total number of hospitalization days and patient count from DialysisData.org [112] to fit a Poisson distribution. For each dialysis patient in the facility, we will simulate his/her annual number of hospitalization days using the distribution, as is often done in the literature [113]. We will test the secondary hypothesis that using Auto-ML will be linked to reduced hospitalization days. If the results from a single simulation run appear too skewed, we will conduct multiple runs and then average their results.

### Ethics Approval

We have already acquired institutional review board approvals from UWM and IH for our study.

## Results

Our paper [35] describes our draft method for automating machine learning model selection. The paper shows that compared to the modern Auto-WEKA automatic selection method [25], on six medical and 21 nonmedical benchmark datasets, our draft method reduced search time by 28-fold, classification error rate by 11%, and standard deviation of error rate due to randomization by 36%, on average. On each of these datasets, our draft method can finish the search process in 12 hours or less on a single computer. The results obtained on the medical datasets are similar to those obtained on the nonmedical datasets. The health care researchers in the Veterans Affairs Salt Lake City Health Care System have used our draft method successfully for a clinical research project [114]. One purpose of this study is to improve the draft method so that it can handle larger datasets more efficiently and effectively.

At present, we are writing Auto-ML’s design document. We intend to finish this study by around the year 2022.

## Discussion

Auto-ML will generalize to various clinical prediction/classification problems, as its design relies on no special property of a specific dataset, patient population, or disease. Auto-ML will be tested on nine modeling problems and datasets, each from a distinct health care system. By providing support for common data models (eg, OMOP [88]) and their linked standardized terminologies adopted by a large number of systems, Auto-ML can be used to construct models if attributes required to solve a problem are accessible in a structured dataset or in one of those common data models. This enables data integration and facilitates building models with data from multiple systems. To help users decide whether any data quality issues need to be handled before modeling, Auto-ML will show the numbers of attribute values outside reasonable ranges and numbers of missing values of nonrepeatedly recorded attributes.

The gaps in scope and accuracy mentioned in the introduction exist in many clinical applications. The principles of our approaches to using incomplete medical data and vast attributes generalize to many other clinical applications beyond the two on care management listed in the introduction.

In summary, our new software is designed to efficiently automate machine learning model selection and temporal aggregation of clinical attributes. By making machine learning feasible with limited budgets and data scientist resources, our new software will help realize value from clinical big data and improve patient outcomes. The models that will be built for the two new modeling problems will help improve care management outcomes.

## Abbreviations

AUC: area under the receiver operating characteristic curve
Auto-ML: Automated Machine Learning
ED: emergency department
EDW: enterprise data warehouse
FEV1: forced expiratory volume in 1 second
HIPAA: Health Insurance Portability and Accountability Act
i2b2: Informatics for Integrating Biology and the Bedside
ICD-9: International Classification of Diseases, Ninth Revision
ICD-10: International Classification of Diseases, Tenth Revision
IH: Intermountain Healthcare
NEDS: Nationwide Emergency Department Sample
OMOP: Observational Medical Outcomes Partnership
OR: odds ratio
PCORnet: National Patient-Centered Clinical Research Network
SQL: Structured Query Language
SUS: System Usability Scale
UWM: University of Washington Medicine
Weka: Waikato Environment for Knowledge Analysis
